# Monitoring clinical trials in infectious diseases

**DOI:** 10.46439/allergy.2.019

**Published:** 2021

**Authors:** David L DeMets, Thomas R Fleming, Susan S Ellenberg

**Affiliations:** 1 University of Wisconsin-Madison, Madison, WI, USA; 2 University of Washington, Seattle, WA, USA; 3 University of Pennsylvania, Philadelphia, PA, USA

## Commentary

In early 2020, the contagious and deadly virus, SARS-CoV-2, was identified. The virus spread rapidly worldwide and was declared a pandemic by the World Health Organization (WHO) in March of 2020 [1]. While many who became infected remained asymptomatic, an alarming number of infected individuals developed severe symptoms, often requiring hospitalization and intensive care, or resulting in death.

Many challenges of conducting clinical trials during a major infectious disease outbreak have been discussed [2–8]. For Covid-19, clinical trials were launched quickly to test repurposed drugs with some biological plausibility for effectiveness against this new virus. Many were relatively small single-site studies, but some were quite large randomized trials and global in nature, allowing reliable and generalizable estimates of efficacy [9–12]. At the same time, government and industry initiated major collaborations to develop and evaluate vaccines to reduce the risk of virologically confirmed symptomatic disease, and to prevent occurrence of severe and fatal illness. Fortunately, several of the vaccines have been shown to be effective receiving regulatory authorization by the Food and Drug Administration [FDA] and the European Medical Agency (EMA), moving us forward toward control of the pandemic [13–15]. Some therapeutics and vaccine studies were done through a clinical trials network, either using an existing infrastructure or by rapidly creating a new one. Whether evaluating a single intervention or using a platform trial design to assess multiple interventions [16], these trials were carefully monitored using independent Data Monitoring Committees (DMCs). In this commentary, we extend the insights and lessons learned for DMCs since an earlier discussion of these issues [8].

The fundamental structure, organization and process for randomized clinical trials (RCTs) and DMCs have been well established for decades. Components of multicenter RCTs typically include a sponsor, a steering committee providing trial leadership, a data coordinating center, a statistical analysis center and a network of clinical sites for participant recruitment. Each component is critical to the success of the trial. Another necessary component of any major Phase III trial is an independent DMC that evaluates emerging data for early safety signals or convincing evidence of benefit [17,18] and the quality of trial conduct, particularly as it would affect trial integrity. These evaluations are guided by the basic ethical principle that trials should not continue longer than necessary to evaluate the new intervention compared to a standard-of-care control [17,18]. In particular, trials can be terminated early due to overwhelming evidence of benefit, a clear lack of benefit, convincing evidence of harm, or logistical issues such as poor accrual leaving little hope of achieving an adequate sample size. DMCs typically operate according to a Charter that outlines responsibilities and operations, its independent and multidisciplinary membership, the need for DMC access on an ongoing basis to emerging data on key efficacy measures as well as safety data, the importance of maintaining confidentiality of emerging data, even of pooled outcome data, [19], to reduce risk of prejudgment, and an interim statistical analysis plan. However, not all contingencies can be anticipated and described in a simple algorithm. Thus, the DMC must have the expertise, wisdom, and experience to adapt to the unexpected [20].

Considerable insights about DMC best practices have been gained from clinical trials conducted since the mid-1980s to address the HIV/AIDS epidemic [21–25] and are relevant to the current Covid-19 pandemic. One such lesson was how to manage the conduct of several simultaneous trials testing multiple potential treatments for HIV-infected patients [26]. Two networks, the AIDS Clinical Trial Group, (ACTG) and the Terry Beirn Community Program for Clinical Research in AIDS, (CPCRA), conducted trials involving multiple sponsors, both federal and industry, with each network using its own data coordinating center and statistical center, but with both networks sharing a common DMC. This single DMC needed to review and evaluate an analysis report for each of the multiple ongoing trials. DMC reports had to be well organized and structured similarly from trial to trial for efficiency and clarity.

The DMC meetings also had to be time-managed carefully to efficiently achieve sufficient discussion of each trial report. While the DMC needed to maintain confidentiality of emerging data, presented in an unblinded manner in their DMC reports during a closed session with only DMC members and the independent statistician in attendance, they also needed direct access to insights from the investigators and sponsors. To accommodate these needs, a major contribution to DMC processes was the creation of open sessions at DMC meetings, where all necessary parties could exchange information about trial design, conduct issues and relevant information external to the trial without sharing any unblinded information about emerging efficacy and safety data [26]. The DMC meetings were structured with a closed session for preliminary unblinded data review, an open session, a second closed session for final unblinded data review and formulation of recommendations for trial continuation, modification or termination and then a final open session for the DMC to relay its recommendations to sponsors and investigators. This was an innovative structure that served the ACTG/CPCRA well; similar structures soon became the de facto standard for most major clinical trials [21–25].

The Covid-19 treatment and vaccine trials were quickly launched with independent DMCs, using the well-established model just described, to review emerging measures of safety and efficacy and to provide oversight on trial progress. Networks, or platform trials, for therapeutics included the ACTT-1 [9], RECOVERY [10], REMAP-CAP [11] and WHO SOLIDARITY Therapeutics Trial [12]. The WHO SOLIDARITY Vaccines Trial, due to open in spring 2021, is the only network trial planned to evaluate vaccine candidates [27]. Other large non-network trials of Covid-19 vaccines have also been conducted [13–15]. Each trial/network has a single DMC to monitor all interventions being studied by the network or platform trial. Over the past year, the experience with Covid-19 treatment and vaccine clinical trials has yielded new insights into DMC best practice, particularly as related to research during disease outbreaks.

Recruitment of participants traditionally has been a challenge for clinical trials, sometimes requiring years to achieve the target sample size, but this has not been the case for most Covid-19 trials. The therapeutic networks recruited thousands of patients for their trials in a few weeks due to the very rapid spread of the pandemic, resulting in large numbers of Covid-19-positive patients eligible for these trials. As was the case in the early days of AIDS trials, when few proven treatments were available, Covid-19 patients were generally eager to participate in trials. Additionally, the unprecedented impact of the pandemic on daily lives resulted in extremely rapid enrollment in vaccine trials, with sample sizes of 30,000–40,000 participants entered within weeks. This speed of recruitment places extreme demands on the components of the trial structure starting with the clinic staff treating or vaccinating subjects, the data collection and analysis staff, as well as the DMC. In some cases, the DMC needed to review detailed DMC reports every few weeks rather than the more traditional frequency of 1–3 times each year per trial.

Large simple trials having minimal data collection, while focusing on the critical data for evaluation of safety and efficacy, is not a new concept [28]. However, for Covid-19 trials, simplifying the traditional data collection was mandatory given the urgent need to evaluate treatments and vaccines. In some instances, data from electronic health records, national registries of mortality and morbidity or insurance reimbursement databases have been used [10]. The large simple trial approach not only has had great utility for registrational trials of Covid-19 therapeutics and vaccines, but also may be valuable for studying remaining safety and efficacy questions for authorized vaccines. For example, in settings where vaccine demand exceeds supply, one might randomize individuals between accelerated vs standard timing of access, or between time of second doses, or between different vaccines, in each instance assessing the influence of viral variants of concern [29,30].

While electronic health records enable rapid collection of efficacy and safety data, the sponsor and DMC must have confidence in the reliability of this source of information. Traditionally, many clinical outcome trials have conducted on-site visits, auditing form by form, item by item to validate the collected data. Often an adjudication committee also will review clinical records to validate the investigator-reported outcome, but this process requires access to clinical records and time for committee review. In the Covid-19 pandemic, delays caused by onsite inspection or event classification are not acceptable. Nevertheless, the DMC must be confident that the data provided for review are sufficiently reliable to justify its recommendations, especially those for trial termination that cannot be reversed. The experience in the GUSTO trial of fibrinolytic treatment in emergent heart attack patients provides some reassurance in this regard. That trial was initially conducted without onsite data verification but later onsite inspection of records found few discrepancies, none of which changed conclusions [31]. This experience suggests that the National Institutes of Health traditional quality assurance strategy of centralized computer editing for implausible values, inconsistencies and problematic patterns of missing data, with “for cause” audits conducted as necessary, may be quite adequate and affords a substantial reduced cost in trial conduct as well as allowing for rapid data flow and the DMC review of very current safety and efficacy data. The FDA has sanctioned such approaches in a recent guidance document [32] However, attention must still be afforded to the discovery of serious data errors or biases and the lack of transparency which have already been experienced in a Covid-19 registry observational study such that publications had to be retracted [33].

Another challenge that DMCs face is how to conduct their meetings. During the Covid-19 pandemic physical travel has not been possible. Telephone-based DMC meetings can be effective if DMC members previously had established an effective working relationship. Fortunately, commonly used communication software such as Zoom [34], BlueJeans [35], Cisco Webex [36] or Microsoft Teams [37] have become a standard approach for DMC meetings during the pandemic. DMC members are able to conduct their meetings more effectively and efficiently when they can see each other and the DMC reports are presented on a shared screen.

Despite the urgent need for treatments and vaccines to deal with Covid-19, all involved in the research efforts—trial sponsors, regulators, investigators and the medical community at large—have strongly supported the conduct of large and rigorously designed and conducted trials of Covid-19 treatments and vaccines, with careful monitoring by DMCs. Although the role of the DMC in the clinical trials process is not well understood, or even recognized, by the general public, the intense interest in the development of treatments and vaccines for Covid19 has led to intense scrutiny of the data monitoring process by many, including the financial and political worlds and the press. For example, during the past year, the DMC for a Covid-19 vaccine trial recommended a pause in recruitment related to a potential safety issue. While that issue was addressed and the trial resumed, the announcement of the pause generated considerable confusion and concern as to the circumstances related to that pause, what that meant regarding the potential for success of the trial, and the identity and role of the “secret committee” called the DMC [38]. This experience illustrates the need for the public to better understand the role of the DMC in the clinical trials process. Perhaps articles in major media explaining the role of DMCs would reach the public most effectively [39].

The HIV/AIDS clinical trial experiences beginning more than 3 decades ago offer another lesson on the importance of broader education about the clinical trials process, including DMCs [26]. In the early years of the ACTG/CPCRA trials, many of the interested parties, including sponsors, investigators, investors and the HIV/AIDS community itself, were not aware of the importance of evidence from randomized clinical trials. Many in the HIV/AIDS community pushed for access to proposed treatments before their true benefits and risks had been reliably evaluated. In time, the HIV/AIDS community became educated about the value of evidence from RCTs and became the strongest proponents of rigorous research methods in evaluating new AIDS/HIV treatments, calling for a focus on important clinical outcomes such as survival and symptomatic progression of disease rather than relying on intermediate or surrogate endpoints. They also recognized the importance of the DMC and the process of interim review. This experience motivates the need for education about clinical trials and DMC processes during the current and any future pandemics.

Despite the morbidity and mortality from the SARS-CoV-2 virus, short cuts in the evaluation of Covid-19 therapeutics and vaccines can result in considerably more harm than good. The DMC has a primary responsibility for safeguarding interests of participants while protecting trial integrity, through monitoring trial progress, evaluating benefit-to-risk considerations, and recommending release of trial results when they become convincing and ready for regulatory review.

Another lesson, while not new, is the value of commonality across data collection and data bases from the multiple trials. Efforts by the Clinical Data Interchange Standards Consortium (CDISC) to develop common data elements has enabled major progress in this regard [40]. Such commonality can be useful to DMCs monitoring trials of Covid-19 treatments and vaccines, since they might wish to probe results from completed trials addressing related questions. The need for commonality is even more critical in a pandemic where time is of the essence.

In rare instances, DMCs from similar ongoing trials have shared limited data to evaluate emerging safety issues [26]. Additionally, DMCs overseeing data from ongoing trials should have timely access to source data from related clinical trials soon after their completion and publication if such data could inform recommendations about the ongoing trial [41,42].

DMCs monitoring treatment and vaccine trials must always operate within the guidance of the ethical principles put forward by documents such as the Declaration of Helsinki and national regulations regarding the conduct of clinical research [43]. DMCs also should be conscious that ethical perspectives vary across the world. Thus, membership of the DMC should reflect the diversity of the patient populations being recruited in both the treatment and vaccine settings. With DMC meetings being held virtually using current communication technology, such diversity of membership can more easily be achieved.

## Conclusions

Substantial progress has been achieved with remarkable speed in the evaluation of treatments and vaccines in the battle against the SARS-CoV-2 virus. Yet considerable needs remain, including pursuit of therapeutics providing greater reductions in the risks of severe disease and mortality, and pursuit of additional vaccines, especially those that would be broadly effective against emerging viral variants and have characteristics that would facilitate mass vaccination campaigns to better address worldwide needs [30].

The lessons learned over this past year need to be carried forward to the generation of new Covid-19 trials. Looking beyond the current Covid-19 challenges, we unfortunately can expect more infectious disease epidemics in coming years. The experience gained in the current pandemic, including best practices for DMCs in public health emergencies, will be extremely valuable in enhancing our ability to achieve timely and reliable results in future trials conducted in such settings.

## References

[1] World Health Organization Declares COVID-19 a “pandemic”: Here is what that means, March 11, 2020: https://time.com/5791661/who-coronavirus-pandemic-declaration/ accessed March 20, 2021

[2] Fleming TR, Ellenberg SS. Evaluating interventions for Ebola: the need for randomized trials. Clinical Trials. 2016 Feb;13(1):6–9. 26768563 10.1177/1740774515616944 PMC4767552

[3] Ellenberg SS, Keusch GT, Babiker AG, Edwards KM, Lewis RJ, Lundgren JD, Rigorous clinical trial design in public health emergencies is essential. Clinical Infectious Diseases. 2018 Apr 17;66(9):1467–9. 29177461 10.1093/cid/cix1032 PMC5905619

[4] Ellenberg SS. Clinical trials in the time of a pandemic. Clinical Trials. 2020 Oct;17[5):467–71. 32650672 10.1177/1740774520939871

[5] Rome BN, Avorn J. Drug Evaluation during the Covid-19 Pandemic. NEJM. 2020 April 14. 10.1056/NEJMp2009457 32289216

[6] Goodman JL, Borio L. Finding Effective Treatments for COVID-19: Scientific Integrity and Public Confidence in a Time of Crises. JAMA 2020 April 16. 10.1001/jama.2020.6434 32297900

[7] Zagury-Orly I, Schwartzstein RM. Covid-19—a reminder to reason. New England Journal of Medicine. 2020 Jul 16;383(3):e12. 10.1056/NEJMp2009405 32343505

[8] DeMets DL, Fleming TR. Achieving effective informed oversight by DMCs in COVID clinical trials. Journal of Clinical Epidemiology. 2020 Oct 1;126:167–71. 32659363 10.1016/j.jclinepi.2020.07.001 PMC7351066

[9] Beigel JH, Tomashek KM, Dodd LE, Mehta AK, Zingman BS, Kalil AC, for the ACTT-1 Study Group. Remdesivir for the Treatment of Covid-19 - Final Report. New England Journal of Medicine. 2020 Nov 5;383(19):1813–1826. 10.1056/NEJMoa2007764 PMC7262788 32445440

[10] RECOVERY Collaborative Group. Dexamethasone in hospitalized patients with Covid-19. New England Journal of Medicine. 2021 Feb 25;384(8):693–704. 10.1056/NEJMoa2021436 PMC7383595 32678530

[11] Investigators REMAP-CAP. Interleukin-6 receptor antagonists in critically ill patients with Covid-19. New England Journal of Medicine. 2021 Apr 22;384(16):1491–502. 10.1056/NEJMoa2100433 PMC7953461 33631065

[12] WHO Solidarity Trial Consortium. Repurposed antiviral drugs for COVID-19—interim WHO SOLIDARITY trial results. New England Journal of Medicine. 2021 Feb 11;384(6):497–511. 10.1056/NEJMoa2023184 PMC7727327 33264556

[13] Polack FP, Thomas SJ, Kitchin N, Absalon J, Gurtman A, Lockhart S, Safety and efficacy of the BNT162b2 mRNA Covid-19 vaccine. New England Journal of Medicine. 2020 Dec 31;383(27):2603–15. 10.1056/NEJMoa2034577 PMC7745181 33301246

[14] Baden LR, El Sahly HM, Essink B, Kotloff K, Frey S, Novak R, Efficacy and safety of the mRNA-1273 SARS-CoV-2 vaccine. New England Journal of Medicine. 2021 Feb 4;384(5):403–16. 10.1056/NEJMoa2035389 PMC7787219 33378609

[15] Sadoff J, Le Gars M, Shukarev G, Heerwegh D, Truyers C, de Groot AM, Stoop J, Tete S, Van Damme W, Leroux-Roels I, Berghmans PJ. Interim Results of a Phase 1–2a Trial of Ad26. COV2. S Covid-19 Vaccine. New England Journal of Medicine. 2021 May 13;384(19):1824–1835. 10.1056/NEJMoa2034201 PMC7821985 33440088

[16] Berry SM, Connor JT, Lewis RJ. The platform trial: an efficient strategy for evaluating multiple treatments. JAMA. 2015 Apr 28;313(16):1619–20. 25799162 10.1001/jama.2015.2316

[17] Ellenberg S, Fleming T and DeMets D: Data Monitoring Committees in Clinical Trials: A Practical Perspective. Second Ed. West Sussex: John Wiley & Sons, Ltd.; 2019.

[18] DeMets DL, Furberg CD, Friedman LM. Data Monitoring in Clinical Trials: A case studies approach. New York: Springer; 2006.

[19] Krause PR, Fleming TR, Ellenberg SS, Henao-Restrepo AM, Krause PR, Fleming T, Maintaining confidentiality of emerging results in COVID-19 vaccine trials is essential. The Lancet. 2020 Nov 21;396(10263):1611–3. 10.1016/S0140-6736(20)32259-5 PMC7834563 33125926

[20] DeMets DL, Ellenberg SS. Data monitoring committees—expect the unexpected. New England Journal of Medicine. 2016 Oct 6;375(14):1365–71. 10.1056/NEJMra1510066 27705256

[21] Calis KA, Archdeacon P, Bain R, DeMets D, Donohue M, Elzarrad MK, Recommendations for data monitoring committees from the Clinical Trials Transformation Initiative. Clinical Trials. 2017 Aug;14(4):342–8. 28503947 10.1177/1740774517707743 PMC5639979

[22] Fleming TR, DeMets DL, Roe MT, Wittes J, Calis KA, Vora AN, Data monitoring committees: promoting best practices to address emerging challenges. Clinical Trials. 2017 Apr;14(2):115–23. 28359194 10.1177/1740774516688915 PMC5380168

[23] Lewis RJ, Calis KA, DeMets DL. Enhancing the scientific integrity and safety of clinical trials: recommendations for data monitoring committees. JAMA. 2016 Dec 13;316(22):2359–60. 27960001 10.1001/jama.2016.16070

[24] Fleming TR, Sharples K, McCall J, Moore A, Rodgers A, Stewart R. Maintaining confidentiality of interim data to enhance trial integrity and credibility. Clinical Trials. 2008 Apr;5(2):157–67. 18375654 10.1177/1740774508089459 PMC2703711

[25] Fleming TR, Ellenberg SS, DeMets DL. Data monitoring committees: current issues. Clinical Trials. 2018 Aug;15(4):321–8. 29629815 10.1177/1740774518764855 PMC6053319

[26] DeMets DL, Fleming TR, Whitley RJ, Childress JF, Ellenberg SS, Foulkes M, The data and safety monitoring board and acquired immune deficiency syndrome [AIDS) clinical trials. Controlled Clinical Trials. 1995 Dec 1;16(6):408–21. 8720018 10.1016/s0197-2456(95)00073-9

[27] WHO. An international randomised trial of candidate vaccines against COVID-19. May, 2020. https://www.who.int/publications-detail/an-international-randomised-trial-of-candidate-vaccinesagainst-covid-19

[28] Yusuf S, Collins R, Peto R. Why do we need some large, simple randomized trials?. Statistics in Medicine. 1984 Oct;3(4):409–20. 6528136 10.1002/sim.4780030421

[29] WHO Ad Hoc Expert Group, Placebo Controlled Trials of Covid-19 Vaccines-Why We Still Need Them, New England Journal of Medicine. Dec 2, 2020.

[30] Krause PR, Fleming TR., Longini IA, Peto R, Briand S, Heymann DL, COVID variants and vaccines: what needs to be done? New England Journal of Medicine. In press.

[31] Eisenstein EL, Collins R, Cracknell BS, Podesta O, Reid ED, Sandercock P, Sensible approaches for reducing clinical trial costs. Clinical Trials. 2008 Feb;5(1):75–84. 18283084 10.1177/1740774507087551

[32] https://www.fda.gov/media/116754/download

[33] Retraction—Hydroxychloroquine or chloroquine with or without a macrolide for treatment of COVID-19: a multinational registry analysis, Lancet, June 4, 2020. https://www.thelancet.com/pdfs/journals/lancet/PIIS0140-6736[20)31324-6.pdf (accessed May 4, 2021). 10.1016/S0140-6736(20)31324-6 PMC7274621 32511943

[34] Zoom Software: https://support.zoom.us, accessed March 20, 2021

[35] Blue Jeans: https://www.bluejeans.com/, accessed March 20, 2021

[36] Web Ex: https://www.cisco.com, accessed March 20, 2021

[37] Microsoft Teams: www.microsoft.com/en-us/microsoft-teams/video-conferencin/ accessed March 20, 2021

[38] Reuters, AstraZeneca pauses coronavirus vaccine trial, rollout doubts dents shares, September 15, 2020. www.reuters.com/article/health-coronavirus-astrazeneca/astrazeneca-pauses-coronavirusvaccine-trial-rollout-doubts-dent-shares-idUSKBN260187

[39] Johnson C, Major coronavirus vaccine trial is paused to investigate unexplained illness: The halt shows that safety protections are working, experts say, Washington Post, Sept 2020. https://www.washingtonpost.com/health/2020/09/09astrazeneca-covidvaccine-safety-trial/(accessed May 4, 2021)

[40] CDISC Standards for Clinical Research: https://www.cdisc.org/standards

[41] Institute of Medicine. Sharing Clinical Trial Data: Maximizing Benefits, MInimizing Risk: National Academies Press; 2015 [Available from: http://www.nap.edu/catalog/18998/sharing-clinical-trialdata-maximizing-benefitsminimizing-risk. 25590113

[42] Lo B, DeMets DL. Incentives for clinical trialists to share data. New England Journal of Medicine. 2016 Sep 22;375(12):1112–5. 10.1056/NEJMp1608351 27653562

[43] Friedman LM, Furberg CD, DeMets DL, Reboussin DM, Granger CB. Fundamentals of clinical trials. 5th edition. New York: Springer; 2015.

